# *Anaplasma phagocytophilum*, Sardinia, Italy

**DOI:** 10.3201/eid1108.050085

**Published:** 2005-08

**Authors:** Alberto Alberti, Maria Filippa Addis, Olivier Sparagano, Rosanna Zobba, Bernardo Chessa, Tiziana Cubeddu, Maria Luisa Pinna Parpaglia, Mauro Ardu, Marco Pittau

**Affiliations:** * Università degli Studi di Sassari, Sassari, Italy;; † University of Newcastle, Newcastle upon Tyne, United Kingdom

**Keywords:** zoonosis, groEL, tick-borne diseases, Anaplasma phagocytophilum, molecular diagnosis

**To the Editor:** *Anaplasma phagocytophilum* (formerly *Ehrlichia phagocytophila*), a tick-transmitted pathogen that infects several animal species, including humans (involved as accidental "dead-end" hosts), is the causative agent of human granulocytic anaplasmosis (HGA). It is a pathogen of veterinary importance responsible for tickborne fever of ruminants and for granulocytic anaplasmosis of horses and dogs (1,2). HGA was first described in the United States in 1994 (2) and is emerging in Europe (3). Although only 2 human cases have been reported in Italy (4), serologic and molecular findings have shown *A. phagocytophilum* infections in dogs and *Ixodes ricinus* ticks (5). Incidence, prevalence, and public impact of HGA and horse granulocytic anaplasmosis are, therefore, unknown for this geographic area. From 1992 to 1996, an average rate of 13.4 cases/year/100,000 inhabitants of tick bite–related fever of unknown etiology has been reported on the island of Sardinia, Italy, which is considerably higher than the corresponding national average value of 2.1 cases/year/100,000 inhabitants. Moreover, 117 cases of tick bite–related fever, whose etiology remains obscure, have been reported from 1995 to 2002 in the central west coast area of the island. Local newspapers occasionally report deaths as a result of tick bites, although no HGA-associated deaths have been documented in Europe.

This study investigated *A. phagocytophilum* in Sardinia. From 2002 to 2004, veterinarians based on the central west coast of the island were instructed to collect EDTA blood samples when a suspected case of tick bite–related fever was found at their clinics. A total of 70 blood samples were collected from 50 dogs and 20 horses that showed tick infestation and symptoms consistent with tickborne disease, such as fever, anorexia, jaundice (only in horses), anemia, myalgia, and reluctance to move. Genomic DNA was extracted from the buffy coat obtained by centrifugation of 2 to 4 mL of blood, as previously described (6). Furthermore, DNA was extracted from 50 *Rhipicephalus sanguineus* ticks removed from 30 dogs. Primers EphplgroEL(569)F (ATGGTATGCAGTTTGATCGC), EphplgroEL(1193)R (TCTACTCTGTCTTTGCGTTC), and EphgroEL(1142)R (TTGAGTACAGCAACACCACCGGAA) were designed and used in combination to generate a heminested polymerase chain reaction (PCR) for the selective amplification of 573 bp of the *groEL* gene of *A. phagocytophilum*. The final 50 μL PCR volume of the first PCR round contained 5 μL of the DNA extraction, primers EphplgroEL(569)F and EphplgroEL(1193)R, and HotMaster Taq DNA polymerase (5u/μL, Eppendorf) according to the manufacturer's basic protocol (Eppendorf AG, Hamburg, Germany). Heminested PCR was performed by using 5 μL of each of the first PCR products and primer EphgroEL(1142)R. To confirm the PCR diagnosis, amplicons were digested with the *Hin*dIII restriction endonuclease (predicted digestion pattern: 3 fragments of 525 bp, 21 bp, and 27 bp). *Anaplasma phagocytophilum* DNA was obtained from strain NCH-1 and used as positive control in PCR reactions. Sequences were obtained by cloning the PCR products into the pCR2.1-TOPO vector (Invitrogen S.R.L., Milan, Italy) and using the ABI PRISM Big Dye Terminator Cycle Sequencing Ready Reaction Kit (Applied Biosystems, Foster City, CA, USA), according to the protocols supplied by the manufacturers. Sequences (AY848751, AY848747) were aligned to the corresponding region of other species belonging to *Rickettsiales* by using ClustalX (7). Genetic distances among species were computed by the Kimura 2-parameters method by using MEGA, and were used to construct bootstrapped neighbor-joining trees (8).

Of 120 DNA samples, 1 tick, 3 dog, and 3 horse samples generated the predicted band of 573 bp representative of the *groEL* gene of *A. phagocytophilum*. *Hin*dIII digestions confirmed PCR diagnosis (Figure A1). Two different *groEL* sequence types were obtained from 1 dog and 1 horse and confirmed by BLAST (http://www.ncbi.nlm.nih.gov/Education/BLASTinfo/information3.html) queries as *A. phagocytophilum groEL* sequences (average identity 99%; average E value = 0), indicating that sequences did not reflect contamination. Bootstrapped neighbor-joining trees confirmed the identity of the new sequences obtained, which are closely related to HGA strains isolated in Europe and the United States (Figure).

The molecular approach applied in this study established *A. phagocytophilum* in an area of Sardinia characterized by a high prevalence of tick bite–related fever in humans and animal species. To our knowledge, this is the first evidence of *A. phagocytophilum* in Sardinian dogs and horses and the first documentation of infection in Italian horses caused by pathogenic strains. Therefore, these findings suggest the emergence of *Anaplasma phagocytophilum* in Italy. *Ixodes ricinus* ticks are indicated as vectors transmitting *A. phagocytophilum* in Europe. Although only 0.3% of 4,086 ticks collected in 72 sites of Sardinia (9) have been identified as *Ixodes*, other tick species are better represented on the island (*Rhipicephalus*, 67.2%; *Haemaphysalis*, 24.1%; *Dermacentor*, 4.9%). *A. phagocytophilum* in 1 *Rhipicephalus sanguineus* could indicate a role of this tick in the epidemiology of HGA. Finally, these data indicate the presence of a potential threat to human and animal health and suggest activation of further epidemiologic surveillance and controls.

**Figure:**
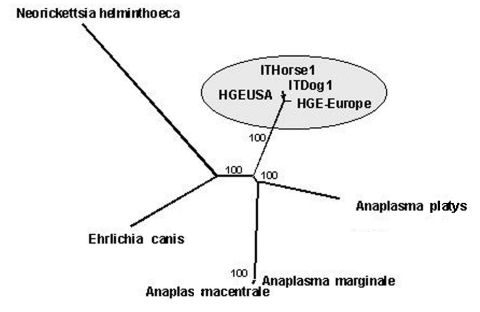
Bootstrapped neighbor-joining tree of several species belonging to Rickettsiales and identification of the strains isolated during the study as *Anaplasma phagocytophilum.* Strains associated to Sardinian *groEL* variants are closely related to European and American pathogenic human granulocytic anaplasmosis strains. Numbers indicate statistically supported bootstrap values.

## Figures and Tables

**Figure A1:**
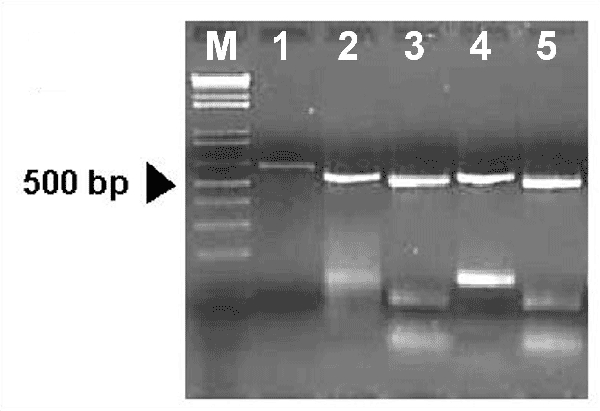
Representative results of groEL heminested polymerase chain reaction (PCR) and HindIII digestion pattern. Lane 1: result of the first PCR round obtained with the strain NCH-1 used as control. Lanes 2, 4: results of the heminested PCR obtained with NCH-1 and with a symptomatic dog, respectively. Lanes 3, 5: HindIII digestions of amplicons shown in lanes 2 and 4, respectively. M:100 bp ladder.
